# The Potential Role of Irisin in Vascular Function and Atherosclerosis: A Review

**DOI:** 10.3390/ijms21197184

**Published:** 2020-09-29

**Authors:** Kyeongho Byun, Sewon Lee

**Affiliations:** 1 Division of Sport Science, College of Arts & Physical Education, Incheon National University, Incheon 22012, Korea; kbyun21@inu.ac.kr; 2 Sport Science Institute, College of Arts & Physical Education, Incheon National University, Incheon 22012, Korea

**Keywords:** vascular function, myokine, exercise, irisin

## Abstract

Exercise is an effective intervention for both the prevention and the treatment of obesity and insulin resistance because skeletal muscle secretes many bioactive proteins that contribute to the beneficial effect of exercise. It has been revealed that irisin plays an important role in metabolic homeostasis and both acute and chronic exercises increase circulating irisin in experimental animal models and in humans. Although previous studies have reported that the irisin-related signaling mechanism may play a beneficial role in the treatment of metabolic diseases including obesity, metabolic syndrome, insulin resistance, and diabetes mellitus, studies on whether irisin plays a key role in vascular function and vascular complications are still insufficient. Therefore, the current review aims to summarize the accumulating evidence showing the potential role of irisin, especially in vascular reactivity and vascular abnormalities such as atherosclerosis.

## 1. Introduction

Exercise is an effective intervention for both the prevention and the treatment of obesity and insulin resistance because skeletal muscle secretes many bioactive proteins that contribute to the beneficial effect of exercise [1,2,3]. Exercise-induced muscle contraction stimulates the gene expression for fibronectin type III domain-containing protein 5 (FNDC5), a membrane protein [1]. The FNDC5 protein is cleaved in skeletal muscles and released into the bloodstream as a polypeptide, which is irisin [1]. It has been revealed that acute and chronic exercise increases circulating irisin both in experimental animal models and in humans [1,4,5,6]. Previous research has shown that the peroxisome proliferator-activated receptor-gamma coactivator-1α (PGC-1α) regulates FNDC5 expression in skeletal muscles [7]. This activation may promote mitochondrion biogenesis, fatty acid β-oxidation, and glucose uptake which enhance energy consumption and stimulate the browning of subcutaneous white adipose tissue (WAT) [1,8]. In this regard, activation of the PGC-1α–FNDC5–irisin axis is an attractive therapeutic target for ameliorating metabolic diseases such as obesity, insulin resistance, and type 2 diabetes mellitus (T2D). Previous studies have shown that metabolic dysfunction decreases circulating irisin levels in experimental obese and diabetic rodent models [9,10], and in humans [11,12]. However, inconsistent studies with these results were also reported. For example, a longitudinal population-based study by Hur et al. indicated that higher circulating irisin levels were related to the development of T2D during a 2.6-year period and circulating irisin levels may be a potential biomarker for the incidence of T2D, regardless of body mass index (BMI) and systemic insulin resistance in Korean adults [13]. In the Hur et al. study, circulating levels of adiponectin, which has anti-diabetic properties was reduced in T2D compared to nonincident diabetic mellitus subjects, suggesting irisin may be associated with incident T2D independently of metabolic risk factors such as BMI and omeostatic model assessment for insulin resistance [13]. Another study by Hur et al. has also shown that circulating irisin levels were comparable between healthy individuals and subjects with metabolic syndrome (MS) even though subjects with MS had higher triglyceride and glucose levels than healthy subjects [14]. In addition, a meta-analysis study suggested that circulating irisin is positively associated with insulin resistance in non-diabetic individuals [15]. Taken together, it seemed that irisin levels were decreased in overt diabetes mellitus but were significantly higher in obesity and metabolic syndrome to compensate metabolic dysfunction similar to insulin resistance in obesity [16,17]. In addition, some previous studies indicated that higher circulating irisin levels were related to cardiovascular diseases (CVD), increased risk of vascular atherosclerosis, and prediction of ischemic stroke [17,18,19,20]. For example, a study by Sesti et al. showed that circulating irisin levels were positively associated with carotid intima-media thickness which was a validated measurement for vascular atherosclerosis, indicating that elevated circulating irisin levels can be a sign of vascular atherosclerosis in a cohort of nondiabetic adult subjects [19]. On the other hand, Li et al. indicated that serum irisin levels were decreased after middle cerebral artery occlusion and exogenous injection of recombinant irisin diminished the brain infarct size in an experimental cerebral ischemia model, suggesting irisin may play an important role in the regulation of neuroprotection [18].

**Figure 1:**
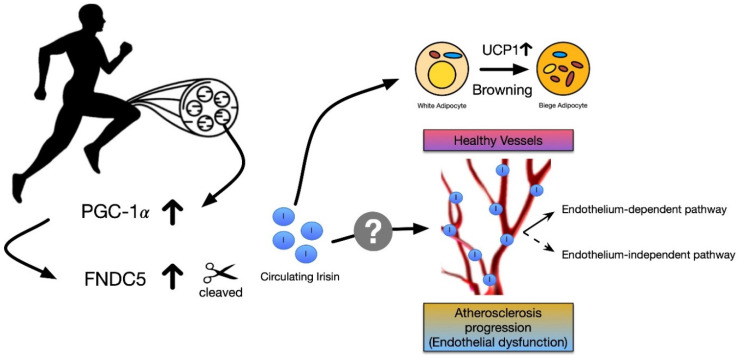
Potential molecular mechanisms underlying effects of irisin secreted by exercising skeletal muscle on vascular function and atherosclerosis. PGC-1α, peroxisome proliferator-activated receptor-gamma coactivator-1α; FNDC5, fibronectin type III domain containing protein 5; UCP1, uncoupling protein 1.

Although previous studies have reported that the irisin-related signaling mechanism plays a positive role in the treatment of metabolic diseases, CVD, and cerebral ischemia, studies on whether irisin plays a pivotal role in vascular function and vascular diseases are still scarce. Therefore, the focus of this review is to provide a comprehensive overview of studies concerning the potential role of irisin in vascular reactivity and atherosclerosis (Figure 1).

## 2. Circulating Irisin Expression in Health and Disease

Initially, irisin has been suggested as a protective exercise-induced hormone against diet-induced obesity, mediated by browning of subcutaneous WAT and thus increased thermogenesis [1]. Subsequent studies have investigated the association of circulating irisin with disease type and condition such as obesity [10,11,12,21], MS [14], diabetes mellitus [9,11,22,23,24,25,26,27,28,29], coronary artery disease (CAD) [30,31], and cerebral ischemia stroke [18] in both human and mouse models. The effect of disease type and condition on circulating irisin concentration has been a matter of considerable debate. A majority of human studies with metabolic dysfunction have shown that lower circulating levels of irisin are associated with obesity [10,12] and T2D [11,23,24,25,26,27,28]. Specifically, it seems obvious that circulating irisin levels are lower in subjects or animal models with diabetes mellitus because a number of studies indicated that serum irisin was reduced in T2D [11,23,24,25,26,27,28], high fat diet- induced T2D mice [9], and streptozotocin-induced type 1 diabetic mice models [29]. However, some studies have also shown that the metabolic disease condition increased circulating irisin in obesity [11,21], MS [17], and T2D [13]. It appears that increased circulating irisin levels are positively associated with BMI and fat mass because compensatory elevated irisin levels act as an anti-obese property to combat metabolic disorders such as obesity, MS, and insulin resistance [16,17,32].

In addition, it is reported that circulating irisin levels are also changed by liver disease. Polyzos et al. showed that serum irisin was higher in lean controls than in non-alcoholic fatty liver and non-alcoholic steatohepatitis patients [33]. Another study by Waluga et al. suggested that serum irisin was higher in control subjects than in alcoholic cirrhosis patients and circulating irisin had significant negative correlation with the grade of inflammation in the non-alcoholic fatty liver disease group [34]. Some studies also have indicated the relationship between irisin, CVD, and cerebrovascular disease. Deng showed that diminished serum irisin levels may be associated with the presence and severity of CAD in human [30] and Hisamatsu et al. suggested higher serum irisin levels were associated with less burden of coronary atherosclerosis assessed by electron-beam computed tomography [31]. A study by Li et al. showed that the serum irisin decreased after cerebral ischemic stroke and circulating irisin levels were negatively associated with brain infarct size, suggesting irisin may contribute to the neuroprotective effect against cerebral ischemia [18].

These discrepant findings imply that the level of circulating irisin is dependent on several factors including the pathological condition, types of disease, production of irisin from skeletal muscles and adipose tissues, and disappearance in circulation. More detailed studies are needed to further understand the effect of the disease condition on the concentration of circulating irisin. Table 1 shows a summary of studies investigating the effect of disease condition on circulating irisin expression in both human and experimental animal models.

## 3. The Potential Role of Irisin in Vascular Reactivity and Atherosclerosis

Vascular dysfunction is a hallmark that is associated with atherosclerosis and occurs in chronic diseases including dyslipidemia, diabetes mellitus, hypertension, cerebrovascular diseases, CAD, and heart failure [39]. Irisin has been reported to be beneficial in energy metabolism in obesity and insulin resistance [1]. In this regard, it is possible that irisin can play an important role in the regulation of vascular function because metabolic dysfunction is considered as a major risk factor for CVD. Previous studies in humans have shown positive correlation between circulating irisin levels and endothelium-dependent vasodilation [12], flow-mediated dilation [24], and coronary atherosclerosis index [30], suggesting irisin may regulate vascular endothelial function. Metabolic dysfunction such as obesity and T2D is associated with endothelial dysfunction characterized by impaired endothelium-dependent vasorelaxation in the experimental rodent models and in humans [12,40,41,42,43,44]. Although exogenous irisin has been suggested to increase energy expenditure and reduce high fat diet-induced obesity and insulin resistance [1], it is still unclear whether exogenous irisin administration ameliorates vascular dysfunction which is induced by abnormal metabolism, and whether irisin may induce vasoconstriction or vasodilation in the vasculature.

Jiang et al. investigated the vasorelaxant effects of irisin on endothelium-dependent and -independent pathways using endothelium-intact and -denuded arteries in myography experiments ex vivo [45]. They reported that irisin (0.1–100 μM) induced both endothelium-dependent and -independent relaxation in dose-dependent manner in isolated second-order mesenteric arteries from male C57BL/6J mice [45]. In addition, Zhang et al. investigated whether recombinant human irisin dilated rat mesenteric artery with or without endothelium, and found that irisin induced vasorelaxation both with and without endothelium in the vasculature [46]. In the study, irisin-induced vasodilation was diminished without endothelium, indicating that both endothelium and smooth muscle cells were involved in irisin-mediated vasorelaxation [46]. Contrary to these findings, Ye et al. suggested irisin induced endothelium-dependent vasodilation but irisin had no effect on endothelium-independent vasodilation in rat mesenteric arteries [47]. It is still unclear whether irisin regulates vascular function through endothelium or both endothelium and smooth muscle cells in the vasculatures.

Other research teams also investigated whether exogenous irisin treatment improved vascular function in aortas from experimental mouse models. For instance, Zhu et al. reported that exogenous administration of irisin (0.5 mg/kg body weight, intraperitoneal injection) for 2 weeks improved acetylcholine (ACh)-induced endothelium-dependent relaxation which was diminished by 8 weeks by high fat diet-induced T2D in the mouse aorta ex vivo [9]. Han et al. also showed that irisin (0.5 μg/g body weight) intraperitoneal injection for 8 weeks promoted endothelium-dependent (ACh-induced) vasorelaxation in obese mouse aorta [10]. These two previous studies showed that endothelial-independent vasorelaxation (sodium nitroprusside-induced) was comparable among groups, suggesting irisin may regulate vascular reactivity through an endothelium-dependent mechanism. In addition, Hou et al. investigated whether exogenous irisin (0.5 μg/g body weight) for 12 weeks ameliorated endothelial dysfunction in perivascular adipose tissue (PVAT) in obesity and showed that irisin enhanced ACh-induced vasorelaxation in the aorta both with and without PVAT [48]. Another study by this group also that showed exogenous irisin administration for 8 weeks improved the anti-contractile properties of PVAT in thoracic aortas from high-fat diet induced obese mice, suggesting irisin may exert a vasorelaxant effect on obesity-induced increased vasoconstriction [49].

Several studies have also suggested that irisin treatment reduced the degree of carotid and aortic atherosclerotic plaque in apolipoprotein E-knock out mice, an atherosclerotic model [50,51,52]. These studies demonstrated that irisin may suppress neointima formation, endothelial injury, and inflammation in the atherosclerotic vasculature, implying a potential role of irisin in treating atherosclerosis [50,51,52]. The findings of the previous studies suggest that irisin may be a therapeutic target for vascular complications and dysfunction induced by metabolic abnormality. On the other hand, several studies have reported that irisin does not affect blood vessels. For instance, Jinjuan et al. reported that irisin had no vasodilatory effect in isolated mesenteric arteries from spontaneously-hypertensive rats (SHR), a hypertensive animal model ex vivo [53]. A study published recently also reported that when a hypertension rat experimental model was treated with irisin for 2 weeks, no hypotensive effect appeared [54]. Therefore, further studies that investigate the effects of irisin on vascular function are necessary. Taken together, it is reported that irisin treatment may play an important role in metabolic dysfunction, but studies on the effects of irisin on vascular diseases and vascular function are insufficient so it is difficult to draw a clear conclusion. Table 2 shows a summary of studies investigating the effects of irisin on vascular function and atherosclerosis.

## 4. Conclusions

It seems that irisin has beneficial effects on metabolic diseases such as obesity, insulin resistance, and T2D. Some animal studies have shown that exercise-induced irisin increases brain-derived neurotrophic factor which is linked to cognitive function and that exogenous administration of recombinant irisin increases cortical bone mass [55,56]. Taken together, irisin may have a salutary health effect on metabolic disease, bone metabolism, and cognitive function. However, it is controversial whether disease condition increases or decreases circulating irisin levels. Therefore, it should be noted that some discrepancies exist among these studies according to the selection of study population, type of disease, selection of enzyme-linked immunosorbent assay (ELISA) kit, and experimental design, including timing of blood sampling. For example, in one study, plasma irisin levels were decreased 1 h after acute moderate-intensity treadmill exercise and peaked 6 h after the exercise, whereas mRNA and protein expression of FNDC5 in gastrocnemius muscle peaked 24 h after the exercise, suggesting a difference in the modulation of the expression of FNDC5 and irisin [5]. In another study, acute high-intensity endurance and heavy strength exercise in humans led to transient increases (peaked at immediately and 1 h after exercise and then decreased until 24 h) in circulating irisin [4]. Furthermore, circulating irisin was detected in wide range of concentrations according to the selection of ELISA kits (30 pg/mL–30 μg/mL, refer to Table 1). Therefore, the difference in the selection of ELISA kits should be taken into account when researchers analyze circulating irisin concentration. There have also been conflicting results regarding potential relationships between circulating irisin concentration and disease condition including obesity and T2D.

An important question from this review was whether irisin directly causes vasodilation or vasoconstriction in macro- and micro-vasculatures, and if so, what mechanisms are involved in the regulation of vascular activity. Several studies by different research groups have shown that irisin causes vasorelaxation in a dose-dependent manner through both endothelium-dependent and -independent mechanisms [45,46]. However, another study by Fu et al. suggested that irisin did not induce direct dilation in small resistant vessels from SHR [53] and a further study showed that irisin only induced endothelium-dependent vasodilation but not endothelium-independent dilation [47]. Studies on the direct vasorelaxant effect of irisin are insufficient to draw a clear conclusion. Therefore, more well-designed studies to investigate the direct vasorelaxant or vasoconstrict effects of irisin in various vascular beds including macro and micro-vasculatures from various experimental animal models are required. Consistently, chronic exogenous injection of irisin improved ACh-induced vasodilation which was endothelium-dependent dilation and decreased vasoconstriction in the segments of mouse aortas ex vivo [9,10,48,49,50]. Thus, this suggests that irisin may play a partial role as a co-factor to ameliorate vascular dysfunction (endothelium-dependent dilation) caused by obesity and T2D. However, whether irisin plays a role in the regulation of vascular function directly in the vasculature are still unclear. As this has potential implications for cardiovascular complications, further investigations are needed to explain mechanisms involved in these signaling pathways.

Taken together, irisin might be a useful agent for treating abnormal vascular function such as hypertension and atherosclerosis. However, more detailed mechanistic studies and in vivo studies are necessary to establish the efficacy of irisin for treating vascular complications and atherosclerosis.

## Figures and Tables

**Table 1:** Alteration of circulating irisin concentration according to disease condition in human and in experimental animal models.

Subject or Animal Model	Disease Condition	Method to Diagnose Disease	Conclusion	Method to Detect Irisin	Refs
Human	Males and females with T2D	WHO criteria - fasting glucose ≥ 126 mg/dL - or HbA1c ≥ 6.5% - or taking antidiabetic medication	- ↑serum irisin (2.378 vs. control, 1.456 μg/mL)	ELISA (Phoenix Pharmaceuticals, EK-067-52)	[13]
Human	Males and females with MS	National Heart, Lung, and Blood Institute/AHA criteria - The presence of at least three of MS risk factors (central obesity, elevated triglyceride, low HDL cholesterol, high fasting glucose or high blood pressure)	- ↑serum irisin (214.4. vs. control, 162.2 ng/mL)	ELISA (Phoenix Pharmaceuticals, EK-067-52)	[17]
Human	Males and females with T2D	- fasting glucose level ≥ 126 mg/dL (7.0 mmol/L) - or 2 h postprandial blood glucose level ≥ 200 mg/dL (11.1 mmol/L) - or HbA1c ≥ 6.5%	- ↓serum irisin (237.06 ± 21.22 vs. control, 377.81 ± 27.16 ng/mL)	ELISA (BioVision)	[11]
Human	Males and females with obesity	BMI ≥ 30 kg/m^2^	- ↑serum irisin (399.84 ± 16.12 vs. control, 340.87 ± 8.40 ng/mL)	ELISA (BioVision)	[11]
Human	Males and females with obesity	BMI ≥ 25 kg/m^2^	- ↓serum irisin (180.5 ± 22.4 vs. control 194.8 ± 19.9 ng/mL)	ELISA (Phoenix Pharmaceuticals)	[12]
Human	CAD	Angiographic evidence of stenosis ≥ 50% in at least one major coronary artery	- ↓ irisin (119.55 vs. control, 146.22 ng/mL)	ELISA (Phoenix Pharmaceuticals)	[30]
Human	Males and females with IGR or T2D	- T2D ∙ fasting plasma glucose ≥ 7.0 mmol/L ∙ 2 h post-challenge plasma glucose ≥ 11.1 mmol/L - IGR ∙ 6.1 mmol/L ≤ fasting plasma glucose ≤ 7.0 mmol/L ∙ 7.8 mmol/L ≤ 2 h post-challenge plasma glucose < 11.1 mmol/L	- No difference in serum irisin (6.75 vs. 7.36 vs. 7.08 ng/mL)	ELISA (Phonenix Pharmaceuticals, EK-067-29)	[22]
Human	Males and females with T2D	means of HbA1c, 8.3 ± 1.9%	- ↓circulating irisin (204 ± 72 vs. control, 257 ± 24 ng/mL)	ELISA (USCN Life Science)	[23]
Human	Males and females with T2D	ADA criteria	- ↓serum irisin (14.12 ± 3.93 vs. control, 28.98 ± 2.56 ng/mL)	ELISA (A Viscera Bioscience)	[24]
Human	Males and females with T2D	ADA criteria	- ↓serum irisin (279 ± 58 vs. control, 263 ± 38 ng/mL)	ELISA (Phoenix Pharmaceuticals)	[25]
Human	Males and females with T2D	WHO criteria - fasting plasma glucose ≥ 126 mg/dL - or 2 h post-bad plasma glucose ≥ 200 mg/dL - or HbA1c ≥ 6.5%	- ↓serum irisin (24.53 ± 3.53 vs. control, 38.86 ± 2.48 pg/mL)	ELISA (USCN Life Science)	[26]
Human	Males and females with T2D	ADA criteria - HbA1c ≥ 7%	- ↓serum irisin (38.06 vs. control, 58.01 ng/mL)	ELISA (Aviscera Bioscience)	[27]
Human	Males and females with T2D	WHO criteria - fasting plasma glucose ≥ 7.0 mmol/l - 2 h post-load plasma glucose ≥ 11.1 mmol/l	- ↓serum irisin (16.24 ± 5.16 vs. controls 24.35 ± 2.76 ng/mL)	ELISA (Aviscera Bioscience)	[28]
Human	Morbidly obese men and women	- 5 women (BW, 128.7 ± 37.1 kg) - 5 men (BW, 158.8 ± 26.9 kg)	- ↑serum irisin (30% higher than normal weight)	ELISA (Phoenix, EK-067-52)	[21]
Human	Male and female subject with acromegaly	GH and IGF-1 concentration	- ↑serum irisin in active acromegaly	ELISA (Sunred Biological Technology, 201-12-5328)	[35]
Human	Male and female subjects with chronic liver disease	Abdominal ultrasound and laboratory tests	- ↓serum irisin in primary biliary cholangitis (5.82 ± 2.41), nonalcoholic fatty liver disease (4.98 ± 2.017) and alcoholic cirrhosis (3.13 ± 1.96) compared to control (29.67 ± 19.9 μg/mL)	ELISA (BioVendor)	[34]
Human	Male and female subjects with obesity or nonalcoholic fatty liver disease	Liver biopsy and NSFLD Activity Score	- ↓serum irisin in obese controls (34.2 ± 2.0), NAFL (31.4 ± 2.8) and NASH (37.9 ± 3.0) compared to lean control (47.3 ± 2.6 ng/mL)	ELISA (Phoenix Pharmaceuticals)	[33]
Human	Male and female patients with atrial fibrillation	Patients hospitalized due to paroxysmal or persistent AF	- No difference in serum irisin	ELISA (BioVendor, RAG018R)	[36]
Human	Male and female patients with subclinical hypothyroidism	Autoimmune thyroiditis and anti-Microsome antibody	- No difference in serum irisin	ELISA (Sunred Biological Technology, 201-12-5328)	[37]
Human	Male with coronary artery calcification	Electron-beam computed tomography	Higher serum irisin were associated with less burden of coronary atherosclerosis.	ELISA (Adipogen, AG-45A-0046EK-k101)	[31]
Human	Patients with ARDS	Chest x-ray or computed tomography and mechanical ventilation	- ↓serum irisin compared to control	ELISA (USCN Life Science)	[38]
Mouse (Male C57 BL/6)	T2D (high-fat diet, 60% fat for 8 weeks)	IPGTT	- ↓serum irisin	ELISA (NeoBioscience Technology)	[9]
Mouse	Obesity (high-fat diet, 50.1% fat for 8 weeks)	BW↑ 26.4 ± 1.8 vs. 35.3 ± 2.6	- ↓serum irisin (29.12 ± 3.04 vs. control, 35.87 ± 3.95 ng/mL)	ELISA (Not specified)	[10]
Mouse (Male C57BL/6J)	Cerebral ischemia stroke (Middle cerebral artery occlusion model)	70% ↓ in blood flow perfusion	- ↓serum irisin (~37 vs. sham ~70 ng/mL)	ELISA (Phoenix)	[18]
Mouse (Male C57/BL6)	T1D (STZ, 35 mg/kg BW + HFD for 8 weeks)	Plasma glucose level by glucose oxidase method	- ↓serum irisin (30.7 ± 3.5 vs. control, 37.4 ± 4.2 ng/mL)	ELISA (Phoenix Pharmaceuticals)	[29]

ADA, American diabetes association; AF, atrial fibrillation; ARDS, acute respiratory distress syndrome; BMI, body mass index; BW, body weight; CAD, coronary artery disease; ELISA, enzyme-linked immunosorbent assay; GH, growth hormone; HbA1c, hemoglobin A1c; HFD, high fat diet; IGF-1, insulin like growth factor 1; IGR, impaired glucose regulation; IPGTT, intraperitoneal glucose tolerance test; MI, myocardial infarction; MS, metabolic syndrome; NAFL, nonalcoholic fatty liver; NSFLD, nonalcoholic fatty liver disease; NASH, nonalcoholic steatohepatitis; STZ, streptozotocin; T1D, type 1 diabetes; T2D, type 2 diabetes; WHO, World Health Organization; ↑, increased; ↓, decreased.

**Table 2:** The role of irisin in vascular function and atherosclerosis, and potential mechanisms involved.

Subject or Animal Model	Disease Condition	Irisin Treatment or Involvement	Conclusion	Vessels Used	Potential Mechanisms Involved	Refs
Human	Males and females with obesity	Correlation of circulating irisin and EDV	Positive correlation (r = 0.388)	Brachial artery	Endothelium-dependent pathway	[12]
Human	Males and females with T2D	Correlation of circulating irisin and FMD	Positive correlation (r = 0.51)	Brachial artery	Endothelium-dependent pathway	[24]
Human	Males and females with CAD	Correlation of circulating irisin and CAI score	Negative correlation (r = −0.340)	Coronary artery	-	[30]
Mice (male C57 BL/6J)	High fat diet (60% fat) for 8 weeks	IP injection 0.5 mg/kg BW Once a day for 2 weeks	↑ACh-mediated relaxation	Aorta	PKC-β/NADPH oxidase and NF-κB/iNOS	[9]
Mice (male C57 BL/6J)	High fat diet (50.1% fat) for 8 weeks to induce T2D	IP injection 0.5 μg/g^−1^∙day^−1^ for 8 weeks	↑ACh-mediated relaxation	Aorta	AMPK-eNOS pathway	[10]
Mice (male C57 BL/6)	High fat diet for 12 weeks to induce obesity	IP injection 0.5 μg/g^−1^∙day^−1^ for 12 weeks	↑ACh-mediated relaxation	Aortas with and without PVAT	HO-1/adiponectin axis	[48]
Mice (male C57BL/6)	High fat diet for 8 weeks to induce obesity	IP injection 0.5 μg/g^−1^∙day^−1^ for 8 weeks Once daily	↓PE-induced vasoconstriction	Aorta	HO-1/adiponectin axis	[49]
Mice (male C57 BL/6J)	10–12 weeks old	Irisin-induced relaxation (0.1~100 μM)	relaxes in dose-dependent manner in endothelium-intact and denuded mesenteric arteries	Mesenteric arteries (2nd order)	NO-cGMP-dependent pathway Voltage-dependent Ca^2+^ channel Intracellular Ca^2+^ release	[45]
Mice (Apo E + STZ)	STZ injected to induce T1D	Tail-vein injection 0.2 μg/g BW for 12 weeks	- ↑ACh-mediated relaxation - ↓Aortic Plaque Area	Aorta	AMPK-PI3K-Akt-eNOS signaling pathway	[50]
Mice (ApoE KO)	Atherosclerosis	IP injection 0.5 μg/g BW for 8 weeks	↓Aortic lesion area	Aorta	ROS-p38 MAPK-NF-κB signaling pathway	[51]
Mice (Male ApoE KO)	High cholesterol diet + partial ligation of the left common carotid artery	IP injection 0.5 μg/kg BW for 4 weeks	↓Carotid lesion area	Carotid artery	ERK signaling pathway(miroRNA126-5p)	[52]
Male SD rats (200–250 g)	-	Irisin-induced relaxation (10 nM~100 μM)	↑Relaxation in dose-dependent manner	Mesenteric arteries (2nd order)	ATP-sensitive K^+^ channel	[46]
Male SD rat	-	- Irisin-induced relaxation (100 nM)	↑Relaxation	Mesenteric arteries	Endothelium-dependent pathway (TRPV4)	[47]
Male Wistar-Kyoto (control) and SHR (hypertension) rats (16–18 weeks old)	Hypertension	3000 ng/mL pre-incubation (1 h)	- ↑ACh-mediated relaxation - ↓PHE-mediated vasoconstriction - No-direct dilation	Mesentery arteries (3rd order)	AMPK–Akt–eNOS–NO signaling pathway	[53]

ACh, acetylcholine; AMPK, 5′ adenosine monophosphate-activated protein kinase; Akt, protein kinase B; ApoE, apolipoprotein E; ATP, adenosine triphosphate; BW, body weight; CAD, coronary artery disease; CAI, coronary atherosclerosis index; cGMP, cyclic guanosine monophosphate; EDV, endothelium-dependent vasodilation; eNOS, endothelial nitric oxide synthase; ERK, extracellular signal-regulated kinase; HO-1, heme oxygenase-1; iNOS, inducible nitric oxide synthase; IP, intraperitoneal; KO, knock out; NADPH oxidase, nicotinamide adenine dinucleotide phosphate oxidase; NF-κB; nuclear factor kappa B; NO, nitric oxide; PE, phenylephrine; PHE, phenylephrine HCl; PI3K, phosphatidylinositol 3-kinase; PKC-β, protein kinase C beta; PVAT, perivascular adipose tissue; p38 MAPK, p38 mitogen-activated protein kinases; ROS, reactive oxygen species; SD, Sprague Dawley; SHR, spontaneously-hypertensive rat; STZ, streptozotocin; TRPV4, transient receptor potential vanilloid subtype 4; T1D, type 1 diabetes; T2D, type 2 diabetes; ↑, increased; ↓, decreased.

## Abbreviations

ACh	acetylcholine
BMI	body mass index
CAD	coronary artery disease
CVD	cardiovascular diseases
ELISA	enzyme-linked immunosorbent assay
FNDC5	fibronectin type III domain containing protein 5
MS	metabolic syndrome
PGC-1α	peroxisome proliferator-activated receptor-gamma coactivator-1α
PVAT	perivascular adipose tissue
SHR	spontaneously-hypertensive rat
T2D	type 2 diabetes mellitus
WAT	white adipose tissue
